# Timely initiation of breastfeeding and associated factors among mothers of infants under 12 months in South Gondar zone, Amhara regional state, Ethiopia; 2013

**DOI:** 10.1186/s13006-018-0160-2

**Published:** 2018-05-02

**Authors:** Liyew Mekonen, Wubareg Seifu, Zemenu Shiferaw

**Affiliations:** 1 College of Medicine and Health Sciences, Public Health Department, Reproductive Health and Nutrition Unit, Jigjiga University, Jigjiga, Ethiopia; 2 College of Medicine and Health Sciences, Public Health Department, Epidemiology and Biostatistics Unit, Jigjiga University, Jigjiga, Ethiopia

**Keywords:** Timely initiation, Breastfeeding, Associated factors, South Gondar, Ethiopia

## Abstract

**Background:**

Timely initiation of breastfeeding is defined as putting the newborn to the breast within one hour of birth. Significant benefits in reducing neonatal mortality and morbidity can be attained with effective promotion of timely initiation of breastfeeding and exclusive breastfeeding during the first months of life. Therefore, this study was conducted to assess timely initiation of breastfeeding and associated factors among mothers in South Gondar, Amhara regional state, Northern Ethiopia.

**Methods:**

A community based cross-sectional study was employed. A multistage stratified sampling technique was used to select the sample of 845 mothers with 97.4% response rate. Moreover, data were collected by face to face interview using a semi structured questionnaire.

**Result:**

The prevalence of timely initiation of breastfeeding was 48.7% (54.7% in urban and 25.1% in rural areas). The odds of initiation of breastfeeding within one hour was higher for urban mothers (Adjusted Odds Ratio [AOR] 2.1; 95% Confidence Interval [CI] 1.4, 3.3), multiparous mothers (AOR 2.8; 95% CI 2.0, 3.8), mothers who had antenatal care (AOR 3.2; 95% CI 2.0, 5.2), mothers delivered in health institution (AOR 3.1; 95% CI 2.2, 4.6) and mothers delivered vaginally (AOR 4.1; 95% CI 1.7, 9.8) than their respective counterparts.

**Conclusion:**

This study depicts the rate of timely initiation of breastfeeding was low in south Gondar zone. Factors which were positively associated with timely initiation of breastfeeding include urban residence, multiparity, having antenatal care, mother deliver in health institution and vaginal mode of delivery. Therefore, South Gondar health office and healthcare providers have to provide breastfeeding information during antenatal care by giving special emphasis to rural and primiparous mothers in which timely initiation of breastfeeding is poorly practiced. Further study is needed to assess the implementation of policies on timely initiation of breastfeeding.

## Background

Timely initiation of breastfeeding is defined as putting the newborn to the breast within one hour of birth [1]. It was one the ten steps to successful breastfeeding on which the Baby Friendly Hospital Initiative was based and launched in 1992 [2]. Substantial benefits in reducing neonatal mortality and morbidity can be achieved with effective promotion of timely initiation of breastfeeding and exclusive breastfeeding during the first months of life [3].

The World Health Organization (WHO) and United Nation Children’s Fund (UNICEF) recommend initiation of breastfeeding within the first hour after birth and exclusive breastfeeding for the first six months followed by continued breastfeeding to age two years or beyond along with appropriate complementary feeding [4]. Despite these recommendations, only 39% of newborns in the developing world are put to the breast within one hour of birth, and only 37% of infants less than six months of age are exclusively breastfed [5].

The Ethiopian government developed national infant and young child feeding guidelines in 2004 and has tried behaviour change communications on breastfeeding. There are considerable variations by region on timely initiation of breastfeeding, 60% in Amhara region where South Gondar Zone is found and the highest in Dire Dawa regions and Harari (90.5% and 89.4%, respectively) [6, 7].

Therefore, this study addressed the issue of timely initiation of breastfeeding practice and associated factors in South Gondar Zone. The finding of this study can provide relevant information for future planning and interventions of appropriate strategies to promote the timely initiation of breastfeeding practices.

## Methods

### Study setting

The study was conducted in South Gondar Zone which is found in Amhara regional state located at 664 km from the capital city Addis Ababa. Based on the 2007 Census conducted by the Central Statistical Agency of Ethiopia, this zone had a total population of 2,051,738, of whom 1,041,061 were men and 1,010,677 were women. A total of 468,238 households were counted in this zone, which results in an average of 4.38 persons to a household. There were 3953 mothers who had children aged less than 12 months at the time of the survey.

### Study design and sample

A community based cross-sectional study was conducted among randomly selected 845 mothers of infants under 12 months in South Gondar Zone, Amhara Regional State, Ethiopia from March to May 2013. Mothers of infants under 12 months who were permanent residents were included. Mothers who were not available during data collection who were unconscious, critically ill and unable to respond were excluded from this study.

### Sample size determination

The required sample size was determined using single population proportion formula with the following assumptions:

- Level of confidence = 95%
- Type I error (α) = 0.05
- 5% margin of error
- Design effect = 2(Multi stage sampling)

Based on the assumption the prevalence of timely initiation of breastfeeding is 50%.

$$ n=\frac{\left(Z\ a/2\right)2\times P\left(1-P\right)}{d2\ } $$

$$ n=\frac{(1.96)2\times 0.5\left(1-o.5\right)}{(0.05)2} $$

$$ n=(384) $$

Given 2 design effect and 10% non-response rate, the final sample size was 845.

### Sampling procedure

A multistage random sampling technique was used for selecting mothers. There were 12 districts in South Gondar zone and from these four districts (Farta district, Estie district, Dera district, Fogera district) were selected by using a simple random sampling technique (lottery method). The sample size was proportionally allocated to the urban and rural kebeles based on the number of mothers who had infants less than 12 months. The sampling frame was prepared for both the urban and rural kebeles by doing census prior to the actual data collection period. Based on the census result there were 718 and 3235 mothers with infants less than 12 month in the rural and urban respectively. Finally 179 and 666 mothers who had infants less than 12 months were selected from rural and urban by using a computer-based generated random number respectively.

### Data collection and instrument

Data were collected using structured and pretested interview questionnaires prepared from Ethiopian Demographic Health Survey and Linkage Project. Data collectors were given two days training for the questionnaires and interviewing techniques. The questionnaires were initially prepared for English and then have been translated into the local language, Amharic and again it was translated back into English to check its accuracy. The questionnaires were pretested and modified before the actual data collection. Four supervisors checked each completed questionnaire and principal investigator monitored the overall quality of the data collection.

### Operational definitions

**Early (timely)** breastfeeding is defined as putting the newborn to the breast within one hour of birth.

**Prelacteal feeding** is feeding to an infant with something other than breast milk after birth to three days whereas ever breastfeeding is defined as mothers breastfeed their index baby.

**Employed mothers** defined as mothers that were employed in governmental, non governmental and private organization.

**Formal education** defined as a person who attended primary or more education.

**No formal education** defined as a person who might able to write or read but did not attend primary or more education.

**Marital status** not in union comprised single, divorced, widow, cohabited and separated.

**Information access** if the mother read books, listen radio or watches a television program.

### Data processing and analysis

Data were cleaned, edited, and entered onto Epidata version 3.2.1 and exported to the statistical packages for social sciences (SPSS) version 20 statistical software for further analysis. Frequency distribution and cross tabulation were done against the variables of interest. Bivariate analyses were done to assess the association between explanatory variables and outcome variable of the study. All variables with a *p* - value of < 0.25 at the bivariate analysis were included into multivariable logistic regression model in which odds ratio with 95% confidence intervals were estimated to identify independent predictors of timely initiation of breastfeeding. A *p* - value less or equal to 0.05 were employed to declare the statistical significance.

## Results

A total of 823 women were participating in this study and making the response rate of 97.4%. The age of the respondents ranged from 15 to 49 with a mean (± SD) age of 27.0 (± 5.7) years. Of the total 823 respondents, 656 (79.7%) were urban dwellers. Majority 765 (93%) of mothers was married, Christian by religion were 690 (83.8%) and 819 (99.5%) were in the Amhara ethnic group. Regarding educational status, 334 (40.6%) of mothers had no formal education, 35% (288) attended primary education and 24.4% (201) respondents attended secondary and higher. Five hundred twelve (62.2%) respondents were housewives. Four hundred and forty-three (53.8%) index infants of mothers were male. The majority of the respondents 572 (69.5) had access to information (Table 1).

**Table 1:** Sociodemographic characteristics of mothers with children less than 12 months in South Gondar Zone, Amhara regional state, Ethiopia, May 2013 (*n* = 823)

Variables	Frequency	Percent
Age of mothers (year)		
< = 20 years	109	13.2
21–34 years	619	75.2
> = 35 years	95	11.5
Residence		
Urban	656	79.7
Rural	167	20.3
Marital status		
Union	765	93.0
Not in union^c^	58	7.0
Religion		
Christian	690	83.8
Muslim	133	16.2
Ethnicity		
Amhara	819	99.5
Tigray	4	0.5
Maternal education		
No formal education	334	40.6
Primary education	288	35.0
Secondary education or higher	201	24.4
Occupation of the mother		
Employed^a^	77	9.4
Unemployed^b^	746	90.6
Child’s sex		
Male	443	53.8
Female	380	46.2
Information access		
Yes	572	69.5
No	251	30.5

^a^Government organization employees, private organization employees

^b^Housewives, daily labourers, farmers, merchants, business owner, students

^c^Single, divorced, widow, cohabited, separated

### Health service related and obstetrics characteristics

The study revealed that 702 (85.3%) mothers had antenatal care during their last pregnancy. Correspondingly, from all mothers were attending antenatal care, 451 (64.2%) mothers were receiving any information related to breastfeeding by healthcare providers. Regarding place of the delivery, the mothers indicated that from the total sampled, the mothers 564 (68.5%) gave birth in a health institution, 362 (64.2%) occurred in the health centre and the rest 202 (35.8%) were in a hospital. Four hundred nineteen (74.3%) had postnatal counselling on breastfeeding among mothers gave birth in health institution.

Among 564 mothers who gave birth in the health institution, 315 (55.9%) bathed their baby after 24 h and 189 (33.5%) within two to 24 h. This study showed that from the 259 (32.0%) mothers who gave birth at home, 157 (60.6%) of them bathed their child within one hour after birth. Three hundred ninety-six (48.1%) of the respondent were para I (primiparous mothers**)** and 189 (23.0%) of them were para II. Regarding to the time of birth, 755 (91.7%) of births were a term and 53 (6.4%) were a post term. Concerning the mode of delivery from all respondents, 795 (96.6%) had a vaginal delivery and 27 (3%) had a caesarean section.

### Breastfeeding related characteristics

Mothers were asked about the time she decided to breastfeed their index child. The response revealed that 505 (61.4%) decided to breastfeed their infants before they became pregnant, 183 (22.2%) decided after delivery and the rest 135 (16.4%) decided during the pregnancy. All mother’s breastfed their index child. Out of all, 808 (98.2%) mothers were breastfeeding their infants at the time of interview. Two hundred and ninety (35.2%) mothers expressed and discarded the first milk (colostrum) before they gave their breast milk for their index child. The most common reason for colostrum expulsion was ‘it is dirty’ 122 (42.1%) followed by, it creates abdominal pain 80 (27.6%) and open the closed nipple 45 (15.5%). One hundred seventy-four (21.1%) mothers introduced prelacteal foods or fluids to their infants. The most common prelacteal food was butter which is reported by 129 (74.1%) of breastfeeding mothers followed by sugar solution 18 (10.3%) and cow milk 15 (8.6%).

### Initiation of breastfeeding

**Fig. 1:**
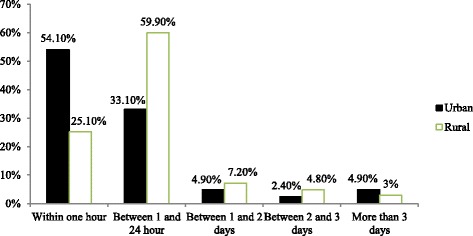
Distribution of breastfeeding initiation by place of residence among mothers in South Gondar zone, Amhara regional state, Ethiopia, May 2013

The proportion of mothers who initiated breastfeeding within one hour differ by residence which was 359 (54.7%) within urban and 42 (25.1%) within rural (Fig. 1).

### Factor influencing timely initiation of breastfeeding

Using multivariate analysis; residence, parity, antenatal care, place of delivery and mode of delivery were identified as an independent predictor for timely initiation of breastfeeding among mothers in South Gondar Zone. Many of independent variables which showed statistical significance with the outcome variables in the bivariate analysis remained significantly associated with outcome variable in multivariate analysis (Tables 2 and 3). In this regard, the odds initiation of breastfeeding within one hour increased for urban mothers (Adjusted Odds Ratio [AOR] 2.1; 95% Confidence Interval [CI] 1.4, 3.3), for multiparous mothers (AOR 2.8; 95% CI 2.0, 3.8), for mothers who had antenatal care (AOR 3.2; 95% CI 2.0, 5.2), among mothers delivered in a health institution (AOR 3.1; 95% CI 2.2, 4.6) and for mothers who delivered vaginally (AOR 4.1; 95% CI 1.7, 9.8) with their respective counterparts (Table 4).

**Table 2:** Bivariate analysis shows the association between sociodemographic characteristics with initiation of breastfeeding among mothers in South Gondar zone, Amhara regional state, Ethiopia, May 2013

Sociodemographic variable	Initiation of breastfeeding			
	≤ 1 h	> 1 h	*p -* value	COR (95% CI)
Age of the mother (years)				
< = 20 years	37 (33.9%)	72 (66.1%)		
20–34 years	320 (51.7%)	299 (48.3%)	0.001	2.1 (1.4, 3.2)
> = 35 years	44 (46.3%)	51 (53.7%)	0.073	1.7 (1.0, 3.0)
Residence				
Urban	359 (54.7%)	297 (45.3%)	0.000	3.6 (2.5, 5.3)
Rural	42 (25.1%)	125 (74.9%)		
Marital status				
Married	373 (48.8%)	392 (51.2%)	0.944	1.0 (0.6, 1.7)
Others	28 (48.3%)	30 (51.2%)		
Maternal education				
Formal education	252 (51.5%)	237 (48.5%)	0.051	1.3 (1.0, 1.8)
No formal education	149 (44.65)	185 (55.4%)		
Religion				
Orthodox	339 (49.3%)	349 (50.7%)	0.477	0.9 (0.6, 1.3)
Others^c^	62 (45.9%)	73 (54.1%)		
Ethnicity				
Amhara	399 (48.7%)	420 (51.3%)	0.959	0.95 (0.1, 6.8)
Tigray	2 (50.0%)	2 (50.0%)		
Occupation of the mother				
Employed^a^	41 (53.2%)	36 (46.8%)	0.405	1.2 (0.8, 1.9)
Unemployed^b^	360 (48.3%)	386 (51.7%)		
Information access				
Yes	312 (54.6%)	259 (45.4%)	0.000	2.2 (1.6, 3.0)
No	89 (35.3%)	163 (64.7%)		
Child’s sex				
Male	214 (48.3%)	229 (51.7%)	0.796	0.96 (0.7, 1.3)
Female	187 (49.2%)	193 (50.8%)		

^a^Government organization employee, private organization employee

^b^Student, daily labourer, housewife, farmer, business owner merchant

^c^Muslim, protestant

**Table 3:** Bivariate analysis shows the association between health service related and obstetrics characteristics with initiation of breastfeeding among mothers in South Gondar Zone, Amhara regional state, Ethiopia, May 2013

Variables	Initiation of breastfeeding			
	≤ 1 h	> 1 h	*p* - value	Crude Odds Ratio (95% CI)
Parity				
Multiparous	238 (55.7%)	189 (44.3%)	0.000	1.8 (1.4, 2.4)
Primiparous	163 (41.2%)	233 (58.8%)		1
Antenatal care				
Yes	370 (52.7%)	332 (47.3%)	0.000	3.2 (2.1, 5.0)
No	31 (25.6%)	90 (74.4%)		1
Time of birth				
Term	369 (48.9%)	386 (51.1%)	0.774	1.1 (0.7, 1.8)
Others^a^	32 (47.1%)	36 (52.9%)		1
Place of delivery				
Health Institution	329 (58.3%)	235 (41.7%)	0.000	3.6 (2.6, 5.0)
Home	72 (27.8%)	187 (72.2%)		1
Mode of delivery				
Vaginal	393 (49.4%)	403 (50.6%)	0.049	2.3 (1.0, 5.4)
Caesarean section	8 (29.6%)	19 (70.4%)		1
Child bathing				
Within one hour	99 (45.6%)	118 (54.4%)	0.287	0.9 (0.6, 1.2)
More than one hour	302 (49.8%)	304 (50.2%)		1

*COR* Crude odds ratio, *CI* Confidence interval, preterm, post term^a^

**Table 4:** Multivariate logistic regression showing factors independently associated with initiation of breastfeeding among mothers in the south Gondar zone, Amhara regional state, Ethiopia, May 2013

Variables	Initiation of breastfeeding			
	≤ 1 h	> 1 h	COR (95% CI)	AOR (95% CI)
Residence				
Urban	359 (54.7%)	297 (45.3%)	3.6 (2.5, 5.3)	2.1 (1.4, 3.3)
Rural	42 (25.1%)	125 (74.9%)	1	1
Parity				
Multiparous	238 (55.7%)	189 (44.3%)	1.8 (1.4, 2.4)	2.8 (2.0, 3.8)
Primiparous	163 (41.2%)	233 (58.8%)	1	1
Antenatal care				
Yes	370 (52.7%)	332 (47.3%)	3.2 (2.1, 5.0)	3.2 (2.0, 5.2)
No	31 (25.6%)	90 (74.4%)	1	1
Place of delivery				
Health institution	329 (58.3%)	235 (41.7%)	3.6 (2.6, 5.0)	3.1 (2.2 4.6)
Home	72 (27.8%)	187 (72.2%)	1	1
Mode of delivery				
Vaginal	393 (49.4%)	403 (50.6%)	2.3 (1.0, 5.4)	4.1 (1.7, 9.8)
Caesarean section	8 (29.6%)	19 (70.4%)	1	1

*COR* Crude odds ratio, *AOR* Adjusted odds ratio, *CI* Confidence Interval

## Discussion

The rate of timely initiation of breastfeeding in this study was 48.7%. It was consistent to findings in Brazil (47.1%) and in Ethiopia Goba Woreda (52.4%) [8, 9]. However this was much lower than findings in Nepal (72.7%), Ethiopia Arjo Woreda 62.6% and south Gondar zone Farta district (76.7%) [10–12]. The prevalence of timely initiation of breastfeeding in this study was (48.7%) higher than in Amhara region (38%) [7].

In this study, 290 (35.2%) respondents expressed and discarded the first milk (colostrum) before starting to breastfeed for their index child. It was consistent with the study conducted in northern part of Ethiopia where colostrum was said to cause abdominal problems, but discarding a portion was sufficient to mitigate this effect [13]. However, according to a study conducted in western Nepal, a significant portion of mothers gave colostrum or breast milk as the first meal to 332 (86.2%) babies, while the remaining 54 (14%) babies were given a fluid other than breast milk as their first feed [10].

This study revealed that 174 (21.1%) mothers were introducing prelacteal foods or fluids to their child. The prevalence of the prelacteal feeding practice in the current study is higher than national findings where the proportion of women who gave prelacteal feeding within the first three days of life was 13% [14]. Another study conducted in the rural northern part of Ethiopia showed that the majority of mothers practiced ritual prelacteal feeding [13]. In addition, in this study, the common prelacteal food introduced to the newborn baby was butter in 129 (74.1%) followed by sugar solution 18 (10.3%) and cow milk in 15 (8.6%). It was consistent with research findings in Mekele town which revealed the common prelacteal food introduced for the newborn babies was butter followed by sugar solution and cow milk [15].

Timely initiation of breastfeeding is influenced by varied and complex interrelated factors and multivariate logistic analysis showed that the odds of timely initiation of breastfeeding among mothers who had antenatal care was increased 3.2 times compared to mothers who had no antenatal care. Correspondingly, mothers that received antenatal care have relative reduced risks of about 8% of delaying breastfeeding initiation than mothers without antenatal care [16]. The possible reason could be that pregnant women who had antenatal care might be informed about timely initiation of breastfeeding by healthcare providers.

The odds of timely initiation of breastfeeding among mothers increased 3.1 times among mothers who had an institutional delivery compared with mothers who delivered at home. A similar study indicated that mothers who delivered their babies at home have an increased relative risk of about 12% of delaying early initiation of breastfeeding than mothers who delivered in the hospitals (or clinics) [16]. This can be explained as mothers who gave birth in health institution had healthcare provider support which helped to initiate breastfeeding timely.

In the current study, the odds of timely initiation increased 4.1 times when mothers delivered vaginally than mothers delivered through caesarean section. It is similar to a study conducted in Nigeria which says mothers that were delivered of their babies through caesarean section have about 58% increased the risk of delaying the early introduction of the first breast milk to their babies as compared to mothers who had a vaginal (normal) delivery [17]. In addition, a systematic review of 53 studies revealed that rates of early breastfeeding (any initiation or at hospital discharge) were lower after caesarean delivery compared with after vaginal delivery [18].

The odds of timely initiation of breastfeeding increased 2.1 times for mothers who resided in an urban than in rural area. Similarly, a study conducted in Ethiopia Goba district showed that urban dwellers were three times more likely to practice timely initiation of breastfeeding when compared to their rural counterparts [9]. In contrast, a study conducted in Al-Hassa province, Saudi Arabia showed that rural mothers were 4.2 times more likely to initiate breastfeeding within one hour [17]. In the current study, the lower rate of timely initiation of breastfeeding in rural areas was probably the traditional practice in the areas; such as early child bathing, colostrum expulsion and prelacteal feeding.

The odds of timely initiation of breastfeeding increased 2.8 times among multiparous mothers than primiparous mothers. In the same way, a study conducted in Turkey showed that breastfeeding initiation was later in primiparous mothers than in mothers who are multiparous [19].

## Conclusions

This study shows that the rate of timely initiation of breastfeeding was low in south Gondar zone. Factors which were positively associated with timely initiation of breastfeeding include urban residence, multiparty, having antenatal care, mother deliver in health institution and vaginal mode of delivery. Therefore, South Gondar health office and healthcare providers have to provide breastfeeding information during antenatal care by giving special emphasis to rural and primiparous mothers where timely initiation of breastfeeding is poorly practiced. Further study is needed to assess the implementation of policies on timely initiation of breastfeeding.

## Abbreviations

AOR: Adjusted Odds Ratio
CI: Confidence Interval
COR: Crude Odds Ratio
SPSS: Statistical Package for Social Science
UNICEF: United Nation Children’s Fund
WHO: World Health Organization
